# Evaluation of secondary malignancies in a large series of mycosis fungoides^☆^

**DOI:** 10.1016/j.abd.2023.06.004

**Published:** 2024-01-22

**Authors:** Tugba Atci, Dilay Yerlioğlu Ak, Can Baykal

**Affiliations:** Department of Dermatology and Venereology, Istanbul Medical Faculty, Istanbul University, Istanbul, Turkey

**Keywords:** Hematologic neoplasms, Lymphomatoid papulosis, Mycosis fungoides

## Abstract

**Background:**

An increased risk of Secondary Malignancies (SMs) in Mycosis Fungoides (MF) has been suggested previously. However, the relationship between this risk and the features of MF is not well-known.

**Objective:**

To investigate the rate and types of SMs in a large cohort of MF patients focusing on the associated features of these patients.

**Methods:**

The demographic features, subtype, and stage of MF, as well as the temporal relationship between the diagnosis of MF and the development of SMs were determined. Major clinical features of MF in this group were compared with MF patients without association of SMs.

**Results:**

Among 730 MF patients with a mean follow-up period of 67.9 ± 52.4 months, 56 SMs were identified in a total of 52 (7.1%) patients. While 28.8% of patients were previously diagnosed with other malignancies, then subsequently had a diagnosis of MF, it was vice versa in 53.8% of patients. Most of the SM-associated MF patients had early-stage (80.7%) and classical type of MF (86.5%) without a significant difference from MF patients without association of SMs; 85.5% and 72.5%, respectively. The most commonly identified SMs were hematologic malignancies (64.3%) including lymphomatoid papulosis (n = 22), Hodgkin’s lymphoma (n = 4), non-Hodgkin’s lymphoma (n = 5), polycythemia vera (n = 2). Other most commonly associated malignancies were breast cancer (n = 4), prostate cancer (n = 3), renal cell carcinoma (n = 2), melanoma (n = 2), and Kaposi’s sarcoma (n = 2).

**Study limitations:**

A single tertiary dermatology center study with a retrospective design.

**Conclusion:**

Apart from the well-known lymphomatoid papulosis association, systemic hematological malignancies were also quite common in the large cohort of MF patients.

## Introduction

Mycosis Fungoides (MF) is the most common type of primary cutaneous lymphoma, classified among the T-cell group of extranodal Non-Hodgkin's Lymphomas (NHL). MF has a rich clinical spectrum of skin lesions. Three MF variants and various clinical presentations have been described. The classical type of MF is the most common presentation, and it is characterized by patches, plaques, and tumors which may be solitary, localized, or generalized. Erythematous, finely scaling patches and plaques with a predilection for the buttocks and other sun‐protected areas represent the early stages of classical MF. Tumoral lesions of MF located anywhere on the body may occur in the late stage of classical MF in only a limited number of patients, but it may also complicate rare types of MF. The rate of visceral involvement increases in MF patients with tumoral lesions. However, most MF patients have early-stage disease throughout their life.^1^, ^2^ Although there are studies suggesting an increased risk of Secondary Malignancies (SMs) in MF patients; particularly systemic lymphomas, factors predisposing to SMs are not described and routine screening for them is still not recommended.^1^, ^2^, ^3^, ^4^, ^5^, ^6^, ^7^, ^8^, ^9^, ^10^, ^11^, ^12^, ^13^, ^14^, ^15^, ^16^, ^17^, ^18^, ^19^, ^20^, ^21^, ^22^, ^23^ The previous studies were limited primarily to either the United States of America (USA)^3^, ^8^, ^9^, ^10^, ^13^, ^15^, ^16^, ^17^, ^18^, ^19^ or Europe (Great Britain and Finland, Denmark, Germany, Poland, Italy),^5^, ^6^, ^7^, ^12^, ^21^, ^22^, ^23^ and scarcely to other geographical regions.^11^, ^14^, ^20^ It is important to investigate the epidemiology of MF-associated malignancies in diverse populations to properly define the pathogenic role of genetic and environmental factors. In addition, the relationship between this risk and the features of MF is not well-known. Herein, the authors aimed to investigate the rate and types of SMs in a large cohort of MF patients and to describe the specific features including subtype and stage of MF in this group of patients, comparing them with features of MF patients without association of SMs.

## Methods

Consecutive MF patients followed up in a single tertiary dermatology center between 2001 and 2023 were retrospectively evaluated regarding the association of SMs. All SMs (excluding cutaneous epithelial tumors) seen in this cohort before, contemporaneously, or after MF diagnosis were included. In addition to the demographic features, subtype, and stage of MF in this group, the temporal relationship between the diagnosis of MF and the development of SMs was determined. Major clinical features of MF in this group were compared with patients without association of SMs. This study was approved by the Institutional Ethical Committee and conducted in accordance with the Declaration of Helsinki (Approval number: E-29624016-050.99-837385).

## Results

Among a cohort of 730 MF patients with a mean follow-up period of 67.9 ± 52.4 months, 56 SMs were identified in a total of 52 (7.1%) patients. Forty-eight patients had one, whereas four patients had two SMs. As 15 (28.8%) patients were previously diagnosed with other malignancy, then subsequently had a diagnosis of MF, it was vice versa in 28 (53.8%) patients, in a mean time of 5.5 ± 5.9 years and 5.2 ± 4.4 years, respectively. In nine (17.3%) patients MF and SMs were diagnosed contemporaneously (Table 1).

**Table 1:** The clinical features of our mycosis fungoides patients and the data regarding secondary malignancy diagnosis.

Sex (number)	Age (mean), (range)^a^	MF subtype	Stage of MF	Secondary malignancy diagnosis	The temporal relationship of secondary malignancy regarding MF diagnosis (years)		
					Before	Contemporaneously	After
**Systemic hematologic malignancies**							
M (n = 4)	43.5 ± 12.5 (27‒57)	Classic (n = 3)	IA (n = 2)	Hodgkin's lymphoma	n = 3 (1‒6)	n = 1	–
		Folliculotropic (n = 1)	IB (n = 1)				
			IVA (n = 1)				
M (n = 3)	58.6 ± 13.5 (40‒74)	Classic (n = 4)	IB (n = 2)	Non Hodgkin’s lymphoma (S-ALCL, n = 4)^c^	n = 1 (3)	–	n = 4 (1‒11)
F (n = 2)		Folliculotropic (n = 1)	IIB (n = 2)				
			IVA (n = 1)				
M (n = 1)	69	Classic + syringotropic	IB	Chronic lymphocytic leukemia	n = 1 (7)	–	–
M (n = 2)	58.5 ± 30.4 (37‒80)	Classic (n = 2)	IB (n = 1)	Polycythemia vera	n = 1 (25)	n = 1	–
			IVA (n = 1)				
M (n = 1)	40	Classic	IB	Hypereosinophilic syndrome	–	–	n = 1 (7)
M (n = 1)	68	Classic	IB	MGUS	n = 1 (5)	–	–
**Primary cutaneous lymphomas**							
M (n = 10)	42.9 ± 15.1 (16‒73)	Classic (n = 19)	IB (n = 12)	Lymphomatoid papulosis	n = 5 (1‒10)	n = 2	n = 15 (1‒20)
F (n = 12)		Folliculotropic (n = 1)	IA (n = 7)				
		Granulomatous (n = 1)	IIA (n = 2)				
		Hyperpigmented (n = 1)	IVA (n = 1)				
		Poikilodermic (n = 1)					
**Cutaneous malignancies**^b^							
M (n = 2)	76.5 ± 2.1 (75‒78)	Folliculotropic (n = 1)	IB (n = 1)	Kaposi’s sarcoma	–	–	n = 2 (1‒3)
		Poikilodermic (n = 1)	IIB (n = 1)				
M (n = 1)	41	Classic	IA	Fibromyxoid sarcoma	–	+	–
M (n = 1)	40 ± 12.7 (31‒49)	Classic (n = 2)	IA (n = 1)	Cutaneous melanoma	n = 1 (12)	‒	n = 1 (9)
F (n = 1)			IIB (n = 1)				
**Solid malignancies**							
F (n = 4)	55.2 ± 16.8 (30‒64)	Classic (n = 4)	IA (n = 2)	Breast cancer	n = 1 (3)	n = 1	n = 2 (2‒7)
			IB (n = 2)				
F (n = 1)	64	Classic	IA	Lung cancer	–	+	–
M (n = 1)	54 ± 8.5 (48‒60)	Classic (n = 2)	IA (n = 1)	Renal cell carcinoma	–	n = 1	n = 1 (1)
F (n = 1)			IIA (n = 1)				
M (n = 3)	67 ± 8.2 (58‒74)	Classic (n = 3)	IB (n = 1)	Prostate cancer	n = 1 (1)	–	n = 2 (8-10)
			IIB (n = 2)				
M (n = 1)	55	Classic	IA	Papillary thyroid cancer	–	n = 1	–
M (n = 1)	55	Classic	IA	Astrocytoma	–	n = 1	–
M (n = 1)	68	Classic	IB	Hepatocellular carcinoma	–	n = 1	–
F (n = 1)	58	Classic	IA	Endometrium cancer	n = 1 (3)	–	–
F (n = 1)	87	Classic	IB	Gastric adenocarcinoma	–	–	n = 1 (2)

M, Male; F, Female; MF, Mycosis Fungoides; MGUS, Monoclonal Gammopathy of Undetermined Significance; S-ALCL, Systemic Anaplastic Large Cell Lymphoma.

a Age at the time of MF diagnosis, years.

b Excluding cutaneous epithelial tumors.

c Associated lymphomatoid papulosis diagnosis in two of them.

The male predominance was present in both groups; 59.6% in SM-associated MF patients and 59.4% in MF patients without association of SMs. The mean age of MF patients at the time of diagnosis of SMs was 51.2 ± 17.1 years which was higher than the mean age of MF diagnosis (43.5 ± 17.8) in the main cohort. Most of the SM-associated MF patients had early-stage MF disease (80.7%) similar to MF patients without association of SMs (85.5%) in the present cohort. Classical type of MF lesions (86.5%) were most common variant in SM-associated MF patients followed by folliculotropic MF (7.7%) compatible with MF patients without association of SMs; 72.5% and 7.5%, respectively (Table 1).

The most commonly identified SMs were hematologic malignancies; 36 malignancies (64.3%) in 34 patients of whom 61.8% were male, including lymphomatoid papulosis (n = 22), Hodgkin’s Lymphoma (HL) (n = 4), NHL (n = 5), polycythemia vera (n = 2), Chronic Lymphocytic Leukemia (CLL) (n = 1), hypereosinophilic syndrome (n = 1) and Monoclonal Gammopathy of Undetermined Significance (MGUS) (n = 1). Other associated malignancies were breast cancer (n = 4), prostate cancer (n = 3), renal cell carcinoma (n = 2), melanoma (n = 2), Kaposi’s sarcoma (n = 2) followed by lung cancer, thyroid cancer, endometrium cancer, cutaneous fibromyxoid sarcoma, hepatocellular carcinoma, gastric adenocarcinoma, and astrocytoma each seen in one patient of whom 55.6% were male (Table 1).

## Discussion

The large series with MF had revealed an association with lymphomatoid papulosis as the most common SM in these patients which has been well-known.^2^, ^24^ Furthermore, various solid neoplasia (n = 15), systemic lymphomas (n = 14), cutaneous sarcomas (n = 3) and cutaneous melanoma (n = 2) were also seen in the present large cohort of MF patients.

The association of MF and SMs has been evaluated in many reports since the 1950s.^3^ Although there are many studies delineating the association of MF and SMs in the literature, the groups included in these studies were not homogeneous which makes it difficult to compare available literature data (Table 2). First of all, some studies included patients only with MF^7^, ^11^, ^12^, ^14^^,^^17^, ^18^, ^19^, ^21^, ^23^ or Sézary syndrome^5^ while others included MF and Sézary syndrome together.^8^, ^10^, ^15^, ^16^^,^^20^, ^22^ Moreover, a few studies referred to their patient group as Cutaneous T-Cell Lymphoma (CTCL)^3^, ^4^, ^6^, ^9^^,^^13^ (Table 2). In addition, some ancient studies primarily focused on cutaneous malignancies due to the possible association with phototherapy applied in the management of MF with limited attention to other SMs.^25^ Moreover, some of the previous studies evaluated the association of MF with only one specific type of SM such as melanoma,^7^ leukemia/lymphoma,^20^ or systemic B-cell lymphomas.^10^ However, a consistent observation in the literature is an increased risk of the development of secondary hematologic malignancies in MF patients, which was also supported by the present study. While 7.1% of MF patients in the current series showed association with SMs (excluding cutaneous epithelial tumors), more than half of them (64.3%) were hematologic malignancies.

**Table 2:** Review of the literature regarding secondary malignancies in mycosis fungoides patients.

Study/Year/Country	Included cutaneous lymphoma types, patient number (n=)	Patients with secondary malignancies, n (%) (non-melanoma skin cancer excluded)	Most common malignancy, n (non-melanoma skin cancer excluded)	Other malignancies (n) (non-melanoma skin cancer excluded)	Ratio of hematologic malignancy among patients with secondary malignancies (%) (non-melanoma skin cancer excluded)
Olsen et al., 1984, USA^3^	CTCL (n = 63)	10 (15.9)	Colon cancer (n = 2)	Bladder cancer (n = 1), prostate cancer (n = 1), pyriform sinus carcinoma (n = 1), thymus germinoma (n = 1), bronchus carcinoma (n = 1), kidney cancer (n = 1), uterus cancer (n = 1), breast cancer (n = 1)	Absent
Kantor et al., 1989, USA^4^	CTCL (n = 544)	35 (6)	Colon cancer (n = 7)	Digestive system cancer (n = 11), respiratory system cancer (n = 11), breast cancer (n = 3), hematologic malignancies (n = 6), others (n = 4)	17.1
Scarisbrick et al., 1999, England^5^	SS (n = 71)	9 (12.7)	Lung cancer (n = 2)	Endometrium cancer (n = 1), ovarian cancer (n = 1), pancreatic cancer (n = 1), HL (n = 1), prostate cancer (n = 1), bladder cancer (n = 1), unknown metastatic (n = 1)	11.1
Väkevä et al., 2000, Finland^6^	CTCL (n = 319)	33 (10)	Lung cancer (n = 12)	Digestive system cancer (n = 5), breast cancer (n = 4), HL (n = 2), NHL (n = 2), genitourinary cancer (n = 2), leukemia (n = 2)	18.2
Evans et al., 2004, United Kingdom^7^	MF (n = 285)	6 (2.1)	Melanoma (n = 6)	Not-included	NA
Huang et al., 2007, USA^8^	MF & SS (n = 429)	37 (8.62)	HL and NHL (n = 7)	Colon cancer (n = 5), lung cancer (n = 5), biliary system cancer (n = 2), melanoma (n = 1)	18.9
Brownell et al., 2008, USA^9^	CTCL (n = 672)	37 (5.5)	NHL (n = 7)	Digestive system cancer (n = 3), respiratory system cancer (n = 4), melanoma (n = 2), HL (n = 2), AML (n = 3), CML (n = 1), multiple myeloma (n = 1), breast cancer (n = 1), prostate cancer (n = 5), female genital cancer (n = 4), urinary system cancer (n = 2), thyroid cancer (n = 1)	37.8
Herro et al., 2009, USA^10^	MF & SS (n = 533)	23 (4.16)	Systemic B-cell lymphomas (n = 23)	Not-included	NA
Hodak et al., 2013, Israel^11^	MF^a^ (n = 343)	51 (15)^a^	Breast cancer (n = 9)^a^ colon and rectal cancer (n = 23)^b^	Hematologic malignancy (n = 16^a^+10^b^), breast cancer (n = 12^a^+9^b^), colon and rectal cancer (n = 23^a^+4^b^), prostate cancer (n = 17^a^+9^b^), lung cancer (n = 6^a^+4^b^), melanoma (n = 7^a^+7^b^), others (n = 7^a^+8^b^)	18.2
	MF^b^ (n = 683)	88 (13)^b^			
Lindahl et al., 2014, Denmark^12^	MF (n = 386)	65 (15)	Urinary tract and genitalia (n = 11)	NHL (n = 8), other hematologic malignancies (n = 2), digestive (n = 10), colorectal cancer (n = 5), respiratory cancer (n = 10), melanoma (n = 1), breast cancer (n = 6)	15.4
Amber et al., 2016, USA^13^	CTCL (n = 2923)	183 (6)	NHL (n = NA)	HL, lung cancer and penile cancer (n = NA)	NA
Cengiz et al., 2017, Turkey^14^	MF (n = 143)	13 (9.1)	HL (n = 3)	NHL (n = 2), adult T-cell leukemia (n = 2), lung cancer (n = 2), renal cell cancer (n = 1), bladder cancer (n = 1), melanoma (n = 1)	53.9
Kommalapati et al., 2018, USA^15^	MF & SS (n = 4774)	472 (10)	NHL and HL (n = NA)	AML, thyroid cancer, prostate cancer, brain cancer, breast cancer, lung cancer (n = NA)	NA
Almukhtar et al., 2019, USA^16^	MF & SS (n = 4229)	550 (13)	NHL (n = 98)	Lung cancer (n = 82), CLL (n = 14), HL (n = 13), melanoma (n = 23)	22.7
Goyal et al., 2020, USA^17^	MF (n = 172)	24 (14)	Melanoma (n = 4)	Anaplastic large cell lymphoma (n = 1), bladder cancer (n = 2), breast cancer (n = 2), CLL (n=1), cholangiocarcinoma (n=1), colon cancer (n = 1) endometrial cancer (1), HL (n = 1), NHL (n = 1) pancreatic cancer (n = 1), prostate cancer (n = 3), renal cancer (n = 3), tongue cancer (n = 1), uterine cancer (n = 1)	12.5
Goyal et al., 2020, USA^18^	MF (n = 6742)	511 (7.5)	NHL (n = 50)	HL (n = 3), leukemia (n = 7), multiple myeloma (n = 2), prostate cancer (n = 19), lung cancer (n = 12), colon cancer (n = 8)	12.1
Goyal et al., 2021, USA^19^	MF (n = 7984)	742 (9.29)	NHL (n = 99)	Melanoma (n = 25), HL (n = 14), lung cancer (n = 69), bladder cancer (n = 20)	15.2
Liu et al., 2021, Canada^20^	MF & SS (n = 5)	5 (NA)	CLL/SLL (n = 5)	Not-included	NA
Scheu et al., 2021, Germany^21^	MF (n = 72)	8 (11)	Hematologic (n = 2)	Solid tumors (n = 5), melanoma (n = 1)	12.5
Błażewicz et al., 2021, Poland^22^	MF & SS (n = 177)	29 (16.4)	Hematologic (n = 12)	Lung cancer (n = 4), breast cancer (n = 2), bladder cancer (n = 2), kidney cancer (n = 2), melanoma (n = 2), others (n = 5)	41.4
Pileri et al., 2021, Italy^23^	MF (n = NA)	13 (NA)	Lung cancer (n = 4)	Prostate cancer (n = 2), pancreatic cancer (n = 2), others (n = 5)	7.7
Present study, 2023, Turkey	MF (n = 730)	56 (7.1)	Hematologic (n = 36)	Breast cancer (n = 4), prostate cancer (n = 3), Kaposi’s sarcoma (n = 2), melanoma (n = 2), renal cell cancer (n = 2), thyroid cancer (n = 1), astrocytoma (n = 1), hepatocellular cancer (n = 1), endometrium cancer (n = 1), fibromyxoid sarcoma (n = 1), lung cancer (n = 1), gastric adenocarcinoma (n = 1)	64.3

AML, Acute Myeloid Leukemia; CTCL, Cutaneous T-cell Lymphoma; CML, Chronic Myeloid Leukemia; CLL, Chronic Lymphocytic Leukemia; GIS, Gastrointestinal System; HL, Hodgkin's Lymphoma; MF, Mycosis Fungoides; NA, Non-Available; NHL, Non-Hodgkin's Lymphoma; SS, Sézary Syndrome; SLL, Small Lymphocytic Lymphoma; USA, United States of America.

a Institution-based cohort.

b Population-based cohort.

Although most of the previous series indicate an increased rate in the association of SMs and MF there are inconsistencies in the type of associated malignancies other than the hematologic ones and the most frequent SMs varied quite a lot (Table 2). In several of these studies, the incidence of SMs was compared with the expected incidence for that population, and more precise data were tried to be obtained.^3^, ^4^, ^6^, ^8^^,^^9^, ^11^, ^12^, ^13^, ^15^, ^17^, ^18^, ^19^, ^21^ However, the main limitations of previous studies are small sample size, short follow-up periods or to be performed from the data of cancer research centers or cancer registries which were not focused on dermatological features of MF (Table 2). Furthermore, in one of database originated studies it was stated that it is not possible to properly investigate the association between NHL and MF due to possible misclassifications of MF as NHL in their database.^11^ The present study represents one of the largest series originating from a single dermatology center. The majority of SM-associated cases had classical MF (86.5%) followed by folliculotropic MF which was similar to the distribution of other patients in the present cohort without association of SM. Whereas the main MF cohort includes nearly 8% pediatric patients, all cases in the SM-associated group were adults. In a large series, the mean age at diagnosis of SM was 61‒72 years, which was 51.2 ± 17.1 years in the studied group with SM association.^19^ A male predominance, like most current studies, was also observed in our SM-associated MF cases (59.6%).^9^, ^13^, ^16^, ^21^

In a previous study conducted by Olsen et al. in 1984, the incidence of SM in CTCL patients was compared with the general population and the overall cancer incidence rate in CTCL patients was 2.4 times, and in white male patients 3.3 times, greater than expected. In addition, a history of prior chemotherapy and a family history of malignancy among first-order relatives were found to be more common among CTCL patients who developed an SM in that study.^3^ However, there was no predominance of any type of SMs and none of them had lymphoproliferative origin in the above-mentioned study. On the contrary, it was remarkable that the literature review cited in this ancient report about the association of MF and SMs between 1950‒1980s’ also showed an association between MF and hematologic malignancies.^3^ Among 544 CTCL patients in a SEER-based study during the period 1973 to 1983 from the USA, SMs developed in 35 (6%) of them, yielding a significantly elevated Relative Risk (RR) of 1.7, which reflects excesses for cancers of the lung, colon, and NHL.^4^ Additionally, the risk of colon cancer increased with a longer duration of follow-up, whereas excesses of lung cancer and NHL were limited to the first 5 years of follow-up of CTCL in the above-mentioned study.^4^ In another study reported from England by Scarisbrick et al. in 1999 in which 71 SS patients were included, it was found that the incidence of internal malignancies in SS patients was twice that reported in patients of similar age treated for HL.^5^

In a recent study based on the data of the Italian Lymphoma Foundation (FIL)-Cutaneous Lymphoma Task Force it was shown that mean modified Severity Weighted Assessment Tool (mSWAT) score before and after SM diagnosis had a significant difference (p = 0.0037) suggesting that MF patients with an SM may have a worse clinical outcome. In the same study, it was hypothesized that by secreting immunosuppressive cytokines or recruiting immunosuppressive cells, a sort of mutual help between the two neoplasms may be prompted.^23^ In addition, before SM onset, early MF patients were mainly observed, like the present study, while only two showed an advanced-stage disease.^23^ In another study conducted by Goyal et al., a cohort of patients with MF was matched with patients with a diagnosis of seborrheic dermatitis by age, sex, and follow-up, and both groups had a pre-existing malignancy diagnosis in the same ratio (10%). In that study, in univariate analysis, patients with MF were significantly more likely to develop an SM than patients with seborrheic dermatitis (Relative Risk [RR] 8.1).^17^

An increased risk for subsequent systemic lymphoma in MF patients was reported in various studies (Table 2). This association may emphasize the yet unknown biological relationship between systemic and cutaneous lymphomas. Contrary to many reports, some studies also investigated the general incidence of primary cutaneous lymphomas in patients with systemic hematological malignancies and it was found that in addition to MF, many other primary cutaneous lymphomas may be seen in this patient group.^20^ Remarkably, 42.3% of SM-associated MF patients had lymphomatoid papulosis diagnosis in our study. A study reporting 23 patients having contemporaneous MF and B-cell malignancies supported that the likelihood of this association is greater than that expected by chance.^10^ In a previous study from Turkey (Istanbul), SMs were diagnosed in 13 (9.1%) of 143 MF patients representing a higher rate than the present study (7.1%). Notably, seven of 13 of these patients (53.8%) had hematologic malignancies, similar to our results (64.3%).^14^ In the present study, early-stage disease (IA-IIA) was seen in 80.7% of MF patients, similar to the rates reported by Cengiz et al.^14^ On the contrary, late-stage disease (stage IIB or higher) was suggested to develop SM significantly more likely in some series, which was not confirmed by this study.^17^ Hodak et al. compared the institution-based cohort including 343 MF patients with 846 age- and gender-matched controls and a total of 683 MF patients in the population-based cohort with 1700 age- and gender-matched controls in their study to examine the association between MF and other malignancies.^11^ In that study from Israel, the ratio of the risk of any cancer of MF patients in both the population-based (13%) and the institution-based cohorts (15%) was higher than in control groups (10% and 9%, respectively). Additionally, it was found that MF and HL, acute leukemia, and lung cancer were strongly associated with each other.^11^ In another study reported from the USA including 4229 MF/SS patients of SEER-13 cancer registries diagnosed between 1994 and 2014, a total of 550 (13%) patients developed an SM with an overall increased risk of 26% compared to the matched general population.^16^ In this study, it was reported that there was a significantly increased risk of HL and NHL in male patients, like the present study, and also an increased risk of melanoma, CLL, HL, NHL, and lung cancer in female patients.^16^ Amber et al. observed that the risk of SM is higher within the first year after MF/SS diagnosis, with an increased risk of HL and NHL in patients >60 and between 20‒39 years, respectively.^13^ High rates of hematological malignancies in these recent studies are further evidence supporting this significant association. In a large cohort of patients with T-cell neoplasms, SMs were developed in 10% of 4774 MF and Sézary syndrome patients, and the increased risk of lung cancer was suggested to point to some biological relationship of those diseases including chromosomal deletions.^15^ In the current study, none of the solid organ neoplasms showed a marked increase. Notably, in most of the patients with associated HL, MF developed afterwards; whereas the NHL was mostly diagnosed during the follow-up period of MF patients. Although it was previously postulated that immune surveillance especially in advanced stages of MF results in a decreased ability for the body to identify and eliminate neoplastic cells, thus not being able to prevent second cancer, most of our patients were in early-stage for whom immunosuppression is not expected.^17^ Early-stage MF patients are treated usually with skin-directed options including topical corticosteroids or phototherapy which were not associated with an increased risk of systemic malignancies.^2^, ^25^ However, one of our melanoma cases had long-term PUVA therapy and both cases with Kaposi’s sarcoma had used topical corticosteroids which may have been triggered by MF therapy.^26^ Interestingly, fibromyxoid sarcoma in one of the patients was not previously reported in MF (Table 2).

The main limitations of the present study are the retrospective nature and to be performed in a single dermatology center.

## Conclusion

Despite the presence of confounding bias in some studies, data in the literature supports an increased risk of developing SM in MF patients. In this cohort, overall malignancy prevalence was not higher than other studies, but the rate of hematologic malignancies was relatively increased which emphasizes the risk of hematologic malignancy developing in MF patients. Furthermore, the patients in the cohort did not show a significant difference in terms of MF subtype and stage compared to other study groups with SMs. Although there is not enough evidence to suggest routine investigation of SMs in all MF patients, extra care should be taken in terms of other hematological diseases if there is a suspicious clinical presentation, laboratory finding, or abnormality in radiological imaging.

## Financial support

None declared.

## Authors’ contributions

Tugba Atci: The study concept and design, data collection, or analysis and interpretation of data, statistical analysis, writing of the manuscript or critical review of important intellectual content, data collection, analysis and interpretation, effective participation in the research guidance, intellectual participation in the propaedeutic and/or therapeutic conduct of the studied cases, critical review of the literature, final approval of the final version of the manuscript.

Dilay Yerlioğlu Ak: Data collection, or analysis and interpretation of data, statistical analysis, writing of the manuscript or critical review of important intellectual content, data collection, analysis and interpretation, effective participation in the research guidance, intellectual participation in the propaedeutic and/or therapeutic conduct of the studied cases, critical review of the literature, final approval of the final version of the manuscript.

Can Baykal: The study concept and design, data collection, or analysis and interpretation of data, statistical analysis, writing of the manuscript or critical review of important intellectual content, data collection, analysis and interpretation, effective participation in the research guidance, intellectual participation in the propaedeutic and/or therapeutic conduct of the studied cases, critical review of the literature, final approval of the final version of the manuscript.

## Conflicts of interest

None declared.
